# Rice (*Oryza sativa L*) plantation affects the stability of biochar in paddy soil

**DOI:** 10.1038/srep10001

**Published:** 2015-05-05

**Authors:** Mengxiong Wu, Qibo Feng, Xue Sun, Hailong Wang, Gerty Gielen, Weixiang Wu

**Affiliations:** 1 Institute of Environmental Science and Technology, Zhejiang University, Hangzhou 310058, PR China; 2 Zhejiang Provincial Key Laboratory for Water Pollution Control and Environmental Safety; 3 School of Environmental and Resource Sciences, Zhejiang A & F University, Lin’an Hangzhou 311300, PR China; 4 Scion, Private Bag 3020, Rotorua, New Zealand

## Abstract

Conversion of rice straw into biochar for soil amendment appears to be a promising method to increase long-term carbon sequestration and reduce greenhouse gas (GHG) emissions. The stability of biochar in paddy soil, which is the major determining factor of carbon sequestration effect, depends mainly on soil properties and plant functions. However, the influence of plants on biochar stability in paddy soil remains unclear. In this study, bulk and surface characteristics of the biochars incubated without rice plants were compared with those incubated with rice plants using a suite of analytical techniques. Results showed that although rice plants had no significant influence on the bulk characteristics and decomposition rates of the biochar, the surface oxidation of biochar particles was enhanced by rice plants. Using ^13^C labeling we observed that rice plants could significantly increase carbon incorporation from biochar into soil microbial biomass. About 0.047% of the carbon in biochar was incorporated into the rice plants during the whole rice growing cycle. These results inferred that root exudates and transportation of biochar particles into rice plants might decrease the stability of biochar in paddy soil. Impact of plants should be considered when predicting carbon sequestration potential of biochar in soil systems.

Biochar is a highly aromatic material that can be produced through pyrolysis of plant biomass (*e.g.* rice straw, grass, wood) or agricultural waste (*e.g.* manure) at low oxygen concentrations and relatively low temperatures. Biochar has received a lot of attention in recent years because of its ability to improve soil fertility and reduce greenhouse gas emissions like nitrous oxide (N_2_O) and methane (CH_4_) from agricultural soils^1^. In addition, due to its relatively high stability, biochar is widely recognized as an important carbon sink in the global carbon cycle, thus decreasing global warming effects^2^,^3^.

The stability of biochar in soil is used as an effective tool to evaluate its carbon sequestration potential. A number of recent studies^4^,^5^,^6^,^7^,^8^ suggest that biochar stability is influenced by the raw material type, the pyrolysis process conditions as well as soil and environmental conditions. Specifically, Zimmerman^9^ showed that biochar degradation decreased with increasing combustion temperatures for a range of biomass materials under different combustion conditions. Hilscher *et al.*^10^ found that mineralization of biochar depended on the type of raw material whereby 3.2% of rye grass-derived biochar and 0.7% of pine-derived biochar were mineralized. Bolan *et al.*^11^ indicated that the decomposition rate of biochar was higher in sandy soils than in clay soils. Nguyen *et al.*^8^ found that mineralization of corn biochar was significantly faster under unsaturated conditions than saturated conditions. Finally, Sigua *et al.*^12^ found that a larger surface enhanced biochar carbon mineralization.

Abiotic and microbial oxidation have been demonstrated as two dominant mechanisms responsible for biochar degradation. Cheng *et al.*^13^ found that during a relatively short-term incubation of 4 months, abiotic oxidation of biochar was more important to increase the potential cation exchange capacity than biotic oxidation. This suggested that abiotic oxidation was very important for the initial oxidation of biochar. Interactions between biochar and microbes are known to occur within a short time period after application to soil, thus influencing the biochar stability in soil^14^. Through a six-month laboratory incubation study, Santos *et al.*^15^ suggested that biochar carbon could be utilized by all bacteria groups, especially gram ( + ) bacteria. Using compound specific isotopic analysis of phospholipid fatty acids, Farrell *et al.*^16^ found that soil microorganisms were able to rapidly take up and metabolize small proportions (3%) of biochar-carbon. In addition, for the direct utilization of biochar carbon within soil microbial communities, microbial degradation of biochar has been shown to occur through co-metabolism. Hamer *et al.*^17^ reported that glucose addition accelerated mineralization of maize biochar by 58%, rye biochar by 72% and wood biochar by 115% via microbial co-metabolism compared to the treatment without glucose addition.

While abiotic and microbial degradation of biochar has been shown to significantly influence biochar stability, the effect of plants on biochar stability is not known. The interaction of plants with the soil and microbes, however, are known to occur through plant roots. Rooting systems provide a source of organic carbon to soil microbes, which enhance microbial biomass, leading to a promotion of microbial co-metabolism that can increase biochar degradation^17^. Roots can also regulate the microbial community structures and activities^18^ by changing soil physical and chemical characteristics (e.g. water and pH) and by exuding organic substances. Therefore, plants are able to directly influence biochar decomposition rates through abiotic or indirectly influence biochar through microbial oxidation. Radial leakage of oxygen from the roots^19^ may enhance oxidation of biochar while organic molecules released from the rice root can adsorb either onto the mineral layers of the biochar or directly to the biochar surfaces^14^. Therefore the adsorption onto biochar contributes to soil organic carbon stabilization and, in turn, plant roots can impact on biochar stability in a soil ecosystem. Especially in paddy fields, the impact of rice plants on biochar stability is expected to occur because paddy soils cycle through saturation and non-saturated states. However, the effects of rice plants on biochar mineralization and characteristics in paddy soil ecosystem remain largely unexplored. Yet, biochar is regularly added to paddy soils because rice-straw derived (RS) biochar applications have proven to be an effective means to reduce greenhouse gas (GHG) emissions, increase carbon sinks and increase crop yields in paddy soils^1^,^20^,^21^,^22^.

The objective of this study was to test the effect of rice plants on the decomposition of RS biochar in paddy soil. We hypothesized that rice plants would stimulate RS biochar degradation via roots exudates and microbial oxidation.

## Results

### Decomposition and characteristics of biochar without rice plants

**Figure 1:**
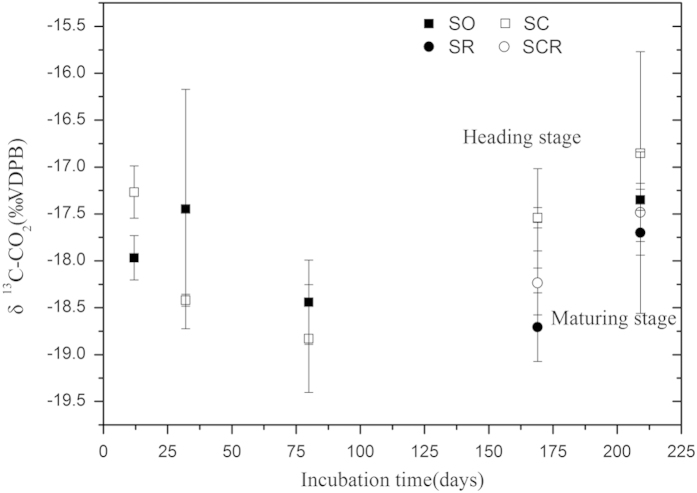
δ^13^C-CO_2_ signature of the evolved CO_2_.Vertiacal bars represent standard deviation of the mean (n = 3).

During the pre-incubation period, the initial CO_2_ efflux rates from the soil were very low. However, the CO_2_ efflux rates increased to a maximum after 12 days of incubation when the rates reached 22.23 mg m^−2^ h^−1^ in the soil without biochar amendment (SO) treatment and 34.56 mg m^−2^ h^−1^ in the biochar amendment (SC) treatment (Fig. S1). After 12 days of pre-incubation, the δ^13^C-CO_2_ evolved from the biochar amendment had a significantly higher signature than that of the control (Fig. 1). As calculated by equation 2, the BC mineralization rate was 2.45 × 10^−2^ μg CO_2_-C g^−1^ biochar h^−1^,or 2.45 × 10^−6^% h^−1^.

### Elemental composition

The C and O compositions of the biochar sampled from the SC treatments after pre-incubation and, at the jointing, heading, and maturing stages of rice growth showed that the total C content increased from 76.28% to 87.20% during the first 3 stages, but decreased during the maturing stage (Table S1). In contrast, O content decreased from the pre-incubation stage until the heading stage and then increased at the maturing stage. As a result, the O/C ratio decreased from 0.24 to 0.08 during the first 3 stages but then increased to 0.13 at the maturing stage.

### Fourier-transform infrared spectra (FTIR) analysis

**Figure 2:**
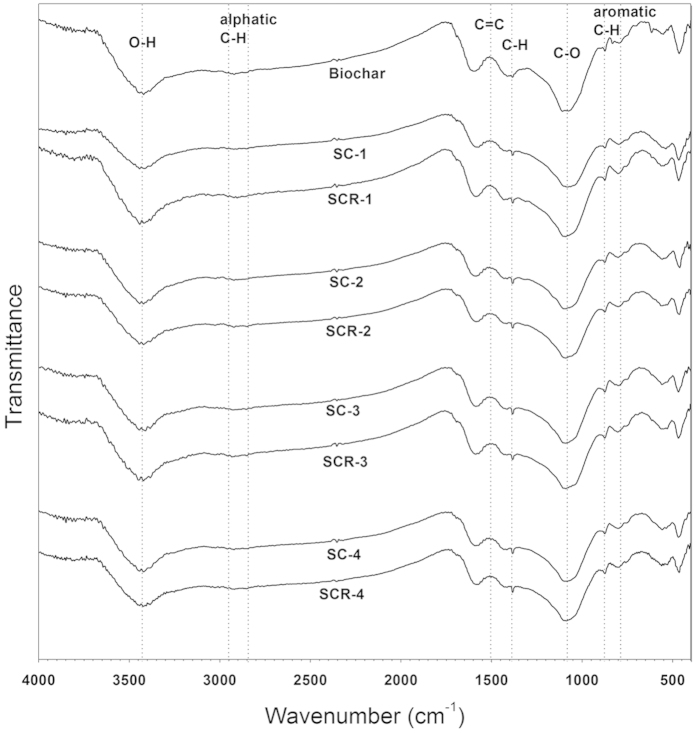
Fourier-transform infrared spectra (FTIR) of the biochar samples collected from different stages of the experiment (biochar: original biochar; −1: pre-incubation;−2: Jointing; −3: Heading; −4: Mature).

Infrared-spectra showed that the original biochar, prior to any treatment, contained carbonyl, hydroxyl and aliphatic C−H groups. After pre-incubation, bands arising from aromatic C-H out of plane vibrations (700–900 cm^−1^) became more apparent in the biochar samples (Fig. 2). Other functional groups, including the aromatic C = C groups (1600 cm^−1^) kept constant during all rice cultivation stages.

### Decomposition and characteristics of biochar during the various rice growth stages

#### Decomposition of biochar

During the rice cultivation stages, CO_2_ efflux rates in the soil were significantly greater in the presence of rice plants than without rice plants, irrespective of biochar amendment. Biochar amendments had no significant impact on the CO_2_ efflux rates in soils during the whole period of rice growth (Fig. S1). During the 4 rice cultivation stages, the CO_2_ efflux rate was constant except during the filling stage when for both treatments with rice plants, the CO_2_ efflux rate was smaller than all other stages.

The δ^13^C−CO_2_ signature of the evolved CO_2_ from biochar decomposition ranged between -19.13‰ and -15.64‰ (Fig. 1). Based on these isotopic CO_2_ signatures, the amount of CO_2_ derived from biochar was determined for the biochar amended treatments at the heading and the maturing stages of the rice growth cycle. Compared to the heading stage, the biochar mineralization rate in the maturing stage, increased when no rice plants were present while the biochar mineralization rate in the biochar treatment with rice plants decreased (Table S2). This resulted in similar biochar mineralization rates during the maturing stage. At the heading and maturing stage, the presence of rice plants did not significantly impact on the biochar mineralization rates.

#### Elemental composition

The C and O compositions of the biochar in the presence of rice plants at the jointing, heading and maturing stages of rice growth showed that the total C content, increased from 85.96% to 86.36% during the first 2 stages of rice growth, but decreased during the maturing stage (Table. S1). In contrast, O content decreased from the jointing stage until the heading stage and then increased at the maturing stage. Therefore, O/C ratio increased from the heading stage until the maturing stage. However, the presence of plants did not significantly impact on the total C content, O content and O/C ratio of biochars sampled from the biochar treatment without (SC) and with (SCR) rice plants during the various rice growing stages.

#### Thermogravimetric (TG) analysis

**Figure 3:**
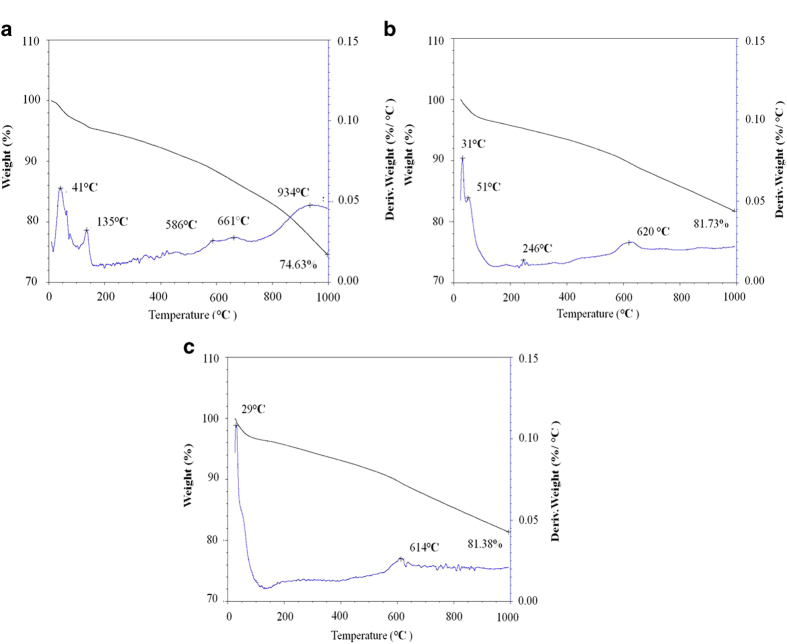
Thermogravimetric (TG) and derivative of thermogravimetric (Dr TG) analysis curves of rice straw-derived biochar (N_2_ flow) (**a**) original biochar; (**b**) biochar from paddy soils with biochar amendment (SC) at the maturing stage; (**c**) biochar from paddy soils with biochar amendment under rice plantation (SCR) at the maturing stage).

The overall weight loss when temperatures were increased to 1000 °C amounted to 25.37% for the original biochar but only 18.27% for the SC treated biochar at the end of the maturing stage and 18.62% for the SCR treated biochar (Fig. 3). The derivative of the thermogravimetric (Dr-TG) analysis curves demonstrated the non-isothermal decomposition of biochar. The greatest rate of weight loss as indicated by Dr-TG analysis curves occurred in the temperature range of 30–60 °C due to moisture losses. When the temperature was greater than 100 °C, the original biochar showed a maximum weight loss rate at 934 °C. Biochar samples from both the SC and SCR treatments had much lower weight loss rates above 750 °C than the original biochar. Biochar samples from both the SC and SCR treatments showed a maximum weight loss rate at 610–620 °C.

#### FTIR analysis

During the whole rice cultivation stage, in the presence of rice plants, functional groups in the biochar samples such as carbonyl, hydroxyl and aromatic C=C (1600 cm^−1^) groups did not show any significant changes over time (Fig. 2). The presence of rice plants did not significantly impact on the functional groups of the biochar samples compared to the samples from the treatments without rice plants.

#### X-ray Photoelectron Spectroscopy (XPS) analysis

The biochar particles and powder after being incubated with rice plants contained significantly more O atoms, as shown by the O1s peak area, than the original biochar and biochar being incubated without rice plants (Fig. S3).

The bulk properties of biochar (biochar powder, Table 1) showed the same trend in elemental contents of C and O as the results of elemental analysis (Table S1), specifically, a small increase in C and a decrease in O after being incubated. The O/C ratio of the original biochar of the interior (biochar powder, Table 1) was similar to the O/C ratio of the surfaces (biochar particles, Table 1). The presence of rice plants did not show a significant difference in the O/C ratio of biochar powder after incubation. However, after incubation with rice plant, the O/C ratio of biochar surfaces increased by 72% of the value of original biochar and 19% of the biochar incubated without rice plants (biochar particles, Table 1). These increases were mainly due to the formation of phenolic C and carboxyl C on the biochar surfaces (Table 2).

#### Incorporation of biochar ^13^C into microbial biomass and rice plants

**Figure 4:**
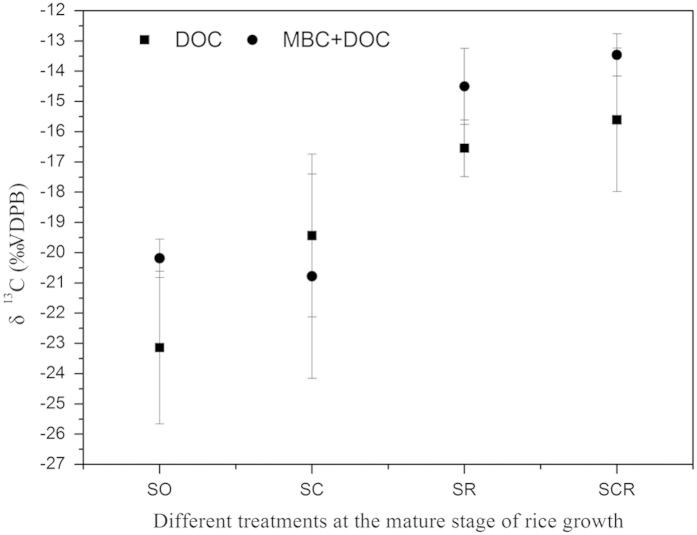
δ^13^-Dissolved organic carbon (DOC) and δ^13^- Dissolved organic carbon plus Microbial biomass carbon (DOC + MBC) of soil at the mature stage of rice growth.

During the tillering, heading and maturing stages of rice growth, the presence of rice plants did not significantly impact on the microbial biomass carbon (MBC) for the biochar amended soils (SC and SCR) (Fig. S2). The isotopic signatures of dissolved organic carbon (DOC) and the sum of DOC and MBC at the maturing stage of rice cultivation in Fig. 4 showed that more ^13^C labeled carbon from the biochar (0.0016% of ^13^C input) was incorporated into the microbial biomass when rice plants were present (SCR) than when they were not (SC) (−0.006% of ^13^C input).

At the end of the maturing stage, the incorporation of ^13^C labeled biochar carbon in the above ground biomass was 0.15% and in the below ground biomass was 0.45% of total ^13^C input (Table 3). In total, 0.047% of the carbon in the biochar was incorporated into rice plants during the whole rice cultivation cycle.

## Discussion

The total thermo-gravimetrical weight loss (25.37%, Fig. 3) of the original biochar was lower than that of the lignin (54.7%) and black carbon-rich soils from natural forests (29.55%)^23^,^24^. Recent studies have shown that TG could be used to estimate the labile fraction of carbon in biochar and evaluate the thermal stability of soil organic matter by using the fraction of volatile matter with respect to the sum of volatile matter content and fixed C^24^,^25^. Therefore, the considerably lower total weight loss in the TG analysis indicated that the biochar used in this study had a relatively high thermal stability.

It has been suggested from the elemental composition that a biochar material containing lower O/C ratios would be more stable than a biochar material with higher O/C ratios^26^. In this experiment, the O/C ratio of the biochars significantly decreased after pre-incubation (Table S1). This suggested that the stability of biochar might be increased after incubation in soils. Mao, *et al.*^27^ showed that biochar residues in *Terra preta* soils were mainly composed of about 6 fused aromatic rings and these biochar residues represented a particularly stable form of soil organic matter. The FTIR in this experiment demonstrated that the intensity of aromatic C-H out-of-plane vibrations (700–900 cm^−1^) of the biochars increased after the pre-incubation period and the aromatic C=C (1600 cm^−1^) functional groups kept constant during the whole incubation period (Fig. 2). This also suggested that the stability of biochar had increased after the pre-incubation period. On the other hand, the total weight loss of the biochar after incubation in soil (SC and SCR treatment) was significantly lower than that of the original biochar. This suggested that the thermal stability of the biochar increased by incubation in soil. Therefore, it could be inferred that biochar after pre-incubation in soil was very stable.

It has been assumed that plants may directly influence biochar decomposition rates by abotic oxidation or indirectly by microbial oxidation. However, in this study rice plants did not have a significant effect on the biochar decomposition rates (Table S2). The method using isotopically labeled CO_2_ has some limitation when it comes to calculating biochar decomposition rates. In particular, biochar could be converted into some forms of organic matter that may be excluded from the calculations. In this experiment, more carbon from biochar was converted to microbial biomass carbon when rice plants were present than when they were not. Kuzyakov, *et al.*^28^ reported that 0.3%0.95% of the ^14^C in biochar was incorporated into microbial biomass and 17% into polysaccharides. These parts of carbon that biochar converted into were not included in the calculations using isotopically labeled CO_2_.

As shown in the FTIR and TG analysis, no significant differences in the functional groups and total weight loss were observed between the biochar samples collected from SC and SCR treatments (Fig. 2, Fig. 3). These results suggested that rice plants had no significant effects on the biochar bulk characteristics. However, the increase of atomic O/C ratios of biochar particles in the presence of rice plants (Table 1) suggested that rice plants enhanced the surface oxidation of the biochar. The differences between surface and bulk properties of the biochars that were exposed to rice plants highlighted the fact that higher oxidative states on the surface region of the biochar particles occurred in comparison to the interior^29^,^30^. Such differences were also observed in this study whereby significant increases in the surface oxygen functional groups of the biochar appeared both in SC and SCR treatments compared to the entire particles (Table 2). Surface oxidation of biochar led to an increase in phenolic groups^13^. In this study, the presence of –COR groups of the biochar significantly increased when biochar was incubated in the presence of rice plants. In addition, LeCroy, *et al.*^31^ reported that biochar exposed to the plant-soil environment reportedly showed more surface oxidation in the form of increased proportions of -COR,-C=O and -COOR groups. Increased surface oxidation of the biochar in the presence of rice plants might have resulted from the rice exudates and radial leakage of oxygen from the roots^19^. Oxygen being the most common electron acceptor, may have oxidised biochar through abotic reactions boosted by a high density of electron donating π-electrons. A short-term incubation conducted by Cheng, *et al.*^13^ at 30 °C with microbial inoculation showed that there were no changes in the measured indicators of surface oxidation, which suggested that surface oxidation might happen as a function of abiotic oxidation, such as chemisorption of oxygen (O) by aging processes^32^. On the other hand, rice exudates, such as low-molecular weight organic compounds (free exudates), high-molecular weight gelatinous materials (mucilage) and sloughed-off cells, could be adsorbed directly onto the biochar surfaces^14^. This would provide more available carbon for soil microorganisms to increase the co-metabolic decomposition of the biochar. Despite that further research is needed to clarify this mechanism, the increase in the biochar surface oxidation of the biochar with rice plants indicated that biochar stability in the soil was influenced by plants in the long term.

Microbes can utilize biochar through two pathways: directly as a carbon source; and indirectly through co-metabolism^17^,^33^. Kuzyakov, *et al.*^34^ indicated that biochar was mainly decomposed through co-metabolism and that biochar was negligible as a microbial C source. In this study, only 0.0016% of carbon from the biochar was incorporated into the soil microbial biomass in the presence of rice plants. The incorporation of the small amount of carbon from biochar into microbial biomass therefore confirms the low microbial availability of biochar as a substrate. However, the amount of carbon from the biochar that was incorporated into the soil microbial biomass in the presence of rice plants was significantly greater than that without the rice plants (−0.006% of ^13^C input, calculated using data from Fig. 4). The enhancement of biochar incorporation into the soil microbial biomass under rice plants may be explained by the effect of root exudates on the microbial co-metabolism in the soil. Root exudates include ions, free oxygen, enzymes, mucilage and a diverse array of carbon-containing primary and secondary metabolites^35^,^36^. These exudates provide a source of organic carbon for soil microbes, leading to a promotion of microbial co-metabolism that can increase the biochar degradation^17^ or enabling some microbes to actively degrade the recalcitrant C compounds with their extracellular enzymes^37^. It is reported that arbuscular mycorrhizal fungi (AMF) could form associations with plant roots through root exudates^38^ and play a pivotal role in facilitating biochar degradation by altering it physically and chemically^39^.

This study has shown a direct incorporation of biochar carbon into rice plants. While only 0.047% of the carbon in the original biochar was incorporated into the rice plant in this study, a large amount of biochar carbon may be lost by being converted to CO_2_ through the metabolism of rice plants in the paddy fields. Especially in China there are many paddy fields. Therefore, the incorporation of biochar carbon into rice plants will greatly decrease the carbon sequestration effect of biochar in a paddy soil ecosystem. Studies have shown that labile components of biochar are susceptible to microbial decomposition. Some metabolites of microorganisms such as acetate and butyrate could also be metabolized by the roots^40^. Therefore, carbon in biochar is not only directly taken up by rice plants but also indirectly utilized by the rice plant through microbial decomposition. Furthermore, it is reported that the transport of biochar particles increased with decreasing particle sizes and pyrolysis temperature^41^. Carbon nanoparticles could be taken up by plant roots, penetrate through vascular systems and then be transported to the plant shoots^42^,^43^. Therefore, we may speculate that rice roots are able to take up some biochar nanoparticles in paddy soil and then transport them into plant shoots. Although the transportation mechanisms of biochar carbon incorporation into rice plants are still not clear, results of this study implied that the role of biochar in carbon sequestration in the soil ecosystem would be greatly overestimated in the long term without the consideration of the impacts of plants. Therefore, the impact of plants should be considered when predicting biochar stability in the soil ecosystem and the role of biochar in the global carbon cycle.

## Methods

### Soil characteristics and production of ^13^C labeled rice-straw biochar

A clay loam soil was collected from a paddy field in Shangyu City, Zhejiang Province, China. In this paddy field, rice plants had been grown under normal rice growing conditions by local farmers. The soil was sampled by taking composite samples in the plowing layer (0–20 cm) of the paddy field and then transported to the agricultural experiment station of Zhejiang University, China where it was air-dried and sieved through a 2-mm mesh screen.

In order to quantify biochar decomposition rates, ^13^C labelled rice straw was generated^44^. The biochar was produced by slowly pyrolysing ^13^C labelled rice straw in a GDL-1500X tubular furnace (Kejin, Hefei, China). The labelled rice straw was heated at a rate of 5–10 °C/min, followed by a residence time of 3 h at 500 °C under a N_2_ gas flow of 1 L/min to ensure an oxygen-free atmosphere. The percentages of ^13^C atoms in the soils and biochars were analyzed by δ^13^C isotope ratio mass spectrometry (ThermoFinnigan/mat 253). The pH of the soils and biochars were measured in a 1:5 (w/ v) and 1:20 (w/ v) water solution (Cheng *et al.*, 2006). Total C, H, N contents were determined using an elemental analyzer (Flash EA1112, Thermo Finnigan, Italy). Ash contents in percent of the biochar were quantified according to the American Society for Testing and Materials (ASTM) D1762-84. Total oxygen contents were calculated by subtracting the C, H and N contents that were water and ash free basis, assuming that biochar and soils were only composed of C, N, H, and O. Detailed physicochemical characteristics of the soils and biochars are summarized in Table 4.

### Experimental setup

Before conducting the experiment, the paddy soil was pre-incubated with (SC treatment) and without ^13^C labeled RS biochar (SO treatment) for 104 days at 20–30 °C with natural light under continuous flooded conditions in plastic buckets (D _b_ = 11 cm, D _t_ = 18.5 cm, H = 10 cm) in order to decompose labile components within the biochar. The Soil-Biochar mixtures were created by incorporating 2.5% (w/w) ^13^C labeled RS biochar into the pre-incubated soil and non-amended soil was used as a control (n = 3 replicates of each).

The pre-incubated soils with and without biochar were used to create 2 experimental treatments (n = 3 replicates of each). These were: Soil with rice plants (SR), and Soil-Biochar mixture with rice plants (SCR). The pre-incubated soils that did not have rice planted in them were used as the Control treatment. All the soil samples were incubated at 20–30 °C with natural light for a complete rice growing season of 105 days. These soils were kept under continuous flooded conditions, and drained 3 weeks prior to the harvest. In order to inhibit the growth of algae, the buckets were shaded with dark foils during the whole incubation period.

Two rice seedlings of the commercially grown variety *Oryza sativa L*., cv. Xiushui 134, were transplanted into each bucket for the treatments with rice plants (SR and SCR). In order to ensure sufficient nutrients for the rice plants to grow, 250 ml of nutrient solution that contained 0.22 g kg^−1^ N (NH_4_NO_3_), 0.20 g kg^−1^ P_2_O_5_ (NaH_2_PO_4_·2H_2_O), 0.20 g kg^−1^ K (K_2_SO_4_) and trace elements, were uniformly mixed with the soils, two weeks before transplanting, and 100 ml of nutrient solution was applied three more times during the whole tillering stage of the rice cultivation stage. Nutrient solutions were also applied to Control soils without rice plants. Enough additional buckets were prepared to enable replicate (n = 3) samples to be destructively sampled at the pre-incubation, tillering, heading and maturing stages of the rice cultivation.

### Carbon dioxide measurements

The CO_2_ flux measurement was determined by the closed chamber method at four growth stages of rice cultivation. These growth stages were the jointing, heading, filling and maturing stages. In order to exclude the influence of photosynthesis, CO_2_ was sampled at night (the sampling lasted for one hour each time). The concentration of CO_2_ in the gas samples was analyzed using a gas chromatograph (GC- 14B, Shimadzu, Japan)^20^ and stable carbon isotopic ratios were measured using ThermoFinnigan/mat253 to estimate the fraction of respired CO_2_ that was derived from biochar.

### Biochar characteristics

Biochar particles (diameters from 150 μm to 300 μm) were randomly picked from the soil of SC and SCR treatments at pre-incubation, jointing, heading and maturing stages using tweezers and an anatomical lens (45 × , SZ61, Olympus)^30^. Elemental compositions of the biochars were conducted by an elemental analyzer (Flash EA1112, Thermo Finnigan, Italy). FTIR absorption spectra were collected from wavelengths 4000 to 400 cm^−1^. In order to prepare biochar samples for FTIR analyses, 1 mg of the samples had been finely ground, sieved through a 150 μm sieve, and mixed with 200 mg KBr. Carbon chemical functional groups were assigned to wave numbers according to reference^21^.

TG and XPS analyses were only conducted on biochar samples collected, from the maturing stage, after 105 days. TG was operated using a AT DSCQ1000 (USA) under a nitrogen atmosphere. The biochar samples, with an initial weight of 10 mg, were placed inside an aluminium oxide crucible. The temperature through the reaction was increased at a heating rate of 10 °C min^−1^. Dr-TG analysis curves were determined.

In order to investigate the surface characteristics of biochar particles, XPS analysis was conducted using a VG ESCALAB MARK II (England), which used a focused monochromatic Mg Kα X-ray (1253 6 eV) source for excitation and a spherical section analyzer. The spectra of the biochar particles were assumed to represent the properties of the particle surface. Finely-ground biochar powder, whose spectra were assumed to represent both exterior and interior properties of the biochar were also measured. The high resolution XPS spectra of C1_S_ and O1_S_ were used to quantify C and O forms on the surface and in the interior of biochar particles. The deconvolution of the C1_S_ and O1_S_ spectra were determined using a non-linear least squares curve fitting program with a Gaussian–Lorentzian mix function and Shirley background subtraction. Details of quantification were described by Cheng *et al.*^13^.

### Soil microbial and plant biomass analyses

Soil samples were collected during the growth season of the rice at 30 days (jointing), 65 days (heading) and 105 days (maturing). Soil MBC was determined on fresh soil samples by the chloroform fumigation-extraction method^45^. Above ground and below ground biomass of the rice plants were collected from each bucket just before harvest, air-dried, ground and sieved through a 0.3 mm mesh screen to measure total carbon with an elementary analyzer (vario MAX, Germany). To quantify the incorporation of ^13^C labeled carbon from biochar into the microbial and rice plants biomass, the ^13^C signature of soil microbial and above ground and below ground biomass carbon at the maturing stage of rice growth was determined by mass spectrometer (ThermoFinnigan/mat253).

### Calculations and statistical analyses

CO_2_ emission fluxes were calculated using the equation: F = ρ × h × dc/dt × 273/T, where F is the CO_2_ emission flux, ρ is the density of CO_2_ at temperature T, h is the height of the closed chamber, dc/dt is the change of the CO_2_ concentration per unit in the closed chamber, and T is the Fahrenheit temperature of the closed chamber.

Isotopic ratios were expressed in standard delta notation as δ^13^CO_2_ in units of per mil (%) relative to the Vienna Pee Dee Belemnite (VPDB) standard. Isotopic ratios are expressed as Atom%^13^C after being labeled, which is calculated as follow (R_Standard_ = 11237.2 ± 90 × 10^−6^):


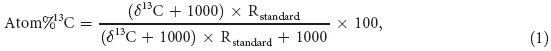


As described by Steinbeiss, *et al.*^46^, the fraction of respired CO_2_, microbial biomass C and plant total carbon (TC) derived from the biochar was calculated as follows:


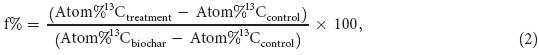


Where Atom%^13^C_treatment_ is the ^13^C atom percentage of different types of carbon with biochar amendment, Atom%^13^C_control_ is the ^13^C atom percentage without biochar amendment, Atom%^13^C_biochar_ is the ^13^C atom percentage of biochar.

All data were reported as means and standard deviation (SD) of the means. Analysis of variance and the least significant difference (LSD) tests were used to determine the significant differences between treatments with and without rice plants at confidence level of P < 0.05.

## Author Contributions

M.X.W. and Q.B.F. designed and performed the experiments. M.X.W wrote the paper with the help of X.S., H.L.W., G.G. and W.X.W. All authors reviewed the manuscript.

## Additional Information

**How to cite this article**: Wu, M. *et al.* Rice (Oryza sativa L) plantation affects the stability of biochar in paddy soil. *Sci. Rep.* **5**, 10001; doi: 10.1038/srep10001 (2015).

## Supplementary Material

Supplementary Information

## Figures and Tables

**Table 1:** Atomic elemental contents of C, O, and atomic O/C ratios measured from X-ray Photoelectron Spectroscopy (XPS) wide scan spectra of biochar particles and finely ground powder (SC: Soil-Biochar mixture, SCR: Soil-Biochar mixture with rice plants).

	**O (%)**	**C (%)**	**O/C (%)**
**Biochar particles**			
Original Biochar	19.9	80.1	0.25
SC	26.5	73.5	0.36
SCR	30.0	70.0	0.43
**Biochar powder**			
Original Biochar	18.6	81.4	0.23
SC	17.9	82.1	0.22
SCR	17.8	82.2	0.22

**Table 2:** Chemical composition of carbon (C1s) and oxygen (O1s) from XPS spectra of biochar particles and finely ground powder.

		**C1s composition (%)**			**O1s composition (%)**	
	**C-C, C=C, or C-H**	**C-O**	**C=O**	**COO**	**O=C**	**-O-H or C-O-C**
**Biochar particles**						
Original Biochar	55.5	24.3	12.6	7.6	35.2	64.8
SC	49.2	24.2	14.1	12.5	42.7	57.3
SCR	49.4	25.2	14.1	11.4	42.7	57.3
**Biochar powder**						
Original Biochar	51.9	24.7	13.7	9.8	40.4	59.7
SC	51.6	24.5	13.2	10.7	44.6	55.4
SCR	50.2	23.6	13.7	12.5	38.3	61.7

The binding energy of C1s at 284.6 eV was assigned to C-C, C=C and C-H, at 286.2 eV to C-O, at 287.4 eV to C=O, and at 289.1 eV to COO. The binding energy of O1s at 531.3 eV was assigned to O=C and at 533.1 eV to O–C.

**Table 3:** Atom%^13^C values of above and below ground biomass of the rice plants for the treatments with and without biochar at the end of the maturing stage.

	**Treatment**	**Total carbon (mg g^-1^)**	**Biomass** (**g**)	**Atom%**^**13**^**C**	**f(%)**	**C**_**biochar**_**(mg)**
Above ground biomass	SR	392.6 ± 1.6a	57 ± 0.75a	1.0797 ± 0.0002b	—	—
SCR	391.8 ± 1.5a	6.47 ± 0.39a	1.0808 ± 0.0002a	0.15	3.86	
Below ground biomass	SR	408 ± 13a	1.25 ± 0.21a	1.0799 ± 0.0001b	—	—
SCR	397.9 ± 5.2a	0.94 ± 0.07a	1.0830 ± 0.0017a	0.45	1.65	

(SR: Soil with rice plants, SCR: Soil-Biochar mixture with rice plants, f indicates the fraction of total carbon derived from biochar calculated by equation 2, C_biochar_ indicates the amount of biochar carbon that was utilized by the rice plants).

**Table 4:** Characteristics of soils and rice-straw derived biochar.

	**Total C (mg g**^**–1**^)	**Total N (mg g**^**–1**^)	**C:N ratio**	^**13**^**C (atom%)**	**pH**
Soil	12.7	1.1	11.5	1.09	8.26
Rice-straw biochar	610.2	22.9	26.6	1.77	10.04
