# Supplementary material for: Rice (Oryza sativa L) plantation affects the stability of biochar in paddy soil
Source: Sci Rep. 2015 May 5;5:10001. doi: 10.1038/srep10001 (PMC4421779; doi:10.1038/srep10001)
Supplement: Supplementary Information [file srep10001-s1.pdf]

Supplementary Information

Rice (*Oryza sativa L*) plantation affects the stability of biochar in paddy soil

Mengxiong Wu[1,2], Qibo Feng[1,2], Xue Sun[1,2], Hailong Wang[3], Gerty Gielen[4], Weixiang Wu[1,2, *]

[1]Institute of Environmental Science and Technology, Zhejiang University, Hangzhou 310058, PR China, [2]Zhejiang Provincial Key Laboratory for Water Pollution Control and Environmental Safety, [3]School of Environmental and Resource Sciences, Zhejiang A & F University, Lin'an Hangzhou 311300, PR China, [4]Scion, Private Bag 3020, Rotorua, New Zealand

Correspondence and requests for materials should be addressed to W.X.W.

(Phone: +86-571-88982020; Fax: +86-571-88982020; E-mail: weixiang@zju.edu.cn.)

Table S1 Elemental analysis of biochar powder during different periods; Values in one column followed by the same letter are not significantly different between SC and SCR treatments at $P < 0.05$ (means ± standard deviation, pooled samples from n=3)

Treatments	Elemental composition（%）		Atom ratio
	C	O	O/C
Original biochar	76.28 ± 0.07b	18.56 ± 0.35	0.24 ± 0.00b
SC (Pre-incubation)	83.67 ± 2.22a	10.44 ± 2.23	0.13 ± 0.03a
SCR (Pre-incubation)	82.32 ± 0.26a	11.97 ± 0.28	0.15 ± 0.00a
SC (Jointing stage)	85.86 ± 0.80a	8.19 ± 0.67	0.10 ± 0.01a
SCR(Jointing stage)	85.96 ± 0.71a	7.87 ± 0.88	0.09 ± 0.01a
SC (Heading stage)	87.20 ± 0.96a	6.80 ± 1.10	0.08 ± 0.01a
SCR (Heading stage)	86.36 ± 1.02a	7.64 ± 0.95	0.09 ± 0.01a
SC (Maturing stage)	83.46 ± 2.88a	10.46 ± 2.83	0.13 ± 0.04a
SCR (Maturing stage)	85.62 ± 2.95a	8.15 ± 3.24	0.10 ± 0.04a

All results were on a dry ash free basis.

Table S2 Biochar derived CO_2 in the soil of the SC and SCR treatments during the Heading and Maturing stages of the rice growth cycle based on ^{13}C isotope (f indicates the fractions of respired CO_2 derived from biochar calculated by equation 2).

	Treatment	CO_2（mg kg^{-1}dw soil）	f (%)	CO_2(μ g g^{-1}biochar h^{-1})
Heading stage	SC	70 ± 24a	0.01	0.23 ± 0.19a
	SCR	376 ± 61b	0.08	11.4 ± 7.2a
Maturing stage	SC	80.0 ± 2.9a	0.16	5.2 ± 4.3a
	SCR	289 ± 58b	0.06	7.4 ± 6.8a

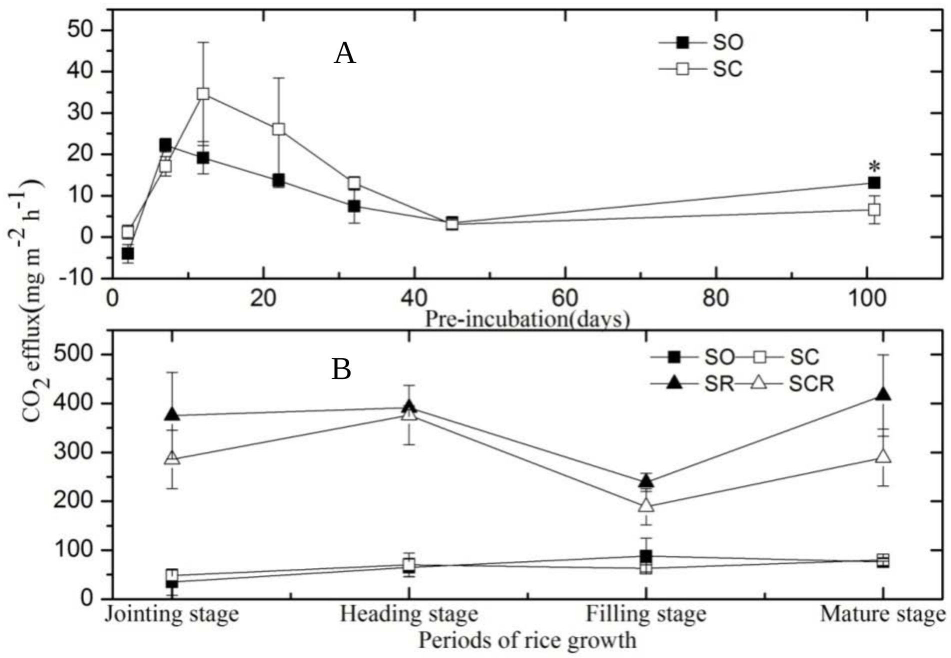

Fig. S1 CO_2 fluxes during pre-incubation time (A) and rice-planting time (B)

("*"indicates significant differences, SO: Soil only, SR: Soil with rice plants, SC: Soil-Biochar mixture, SCR: Soil-Biochar mixture with rice plants)

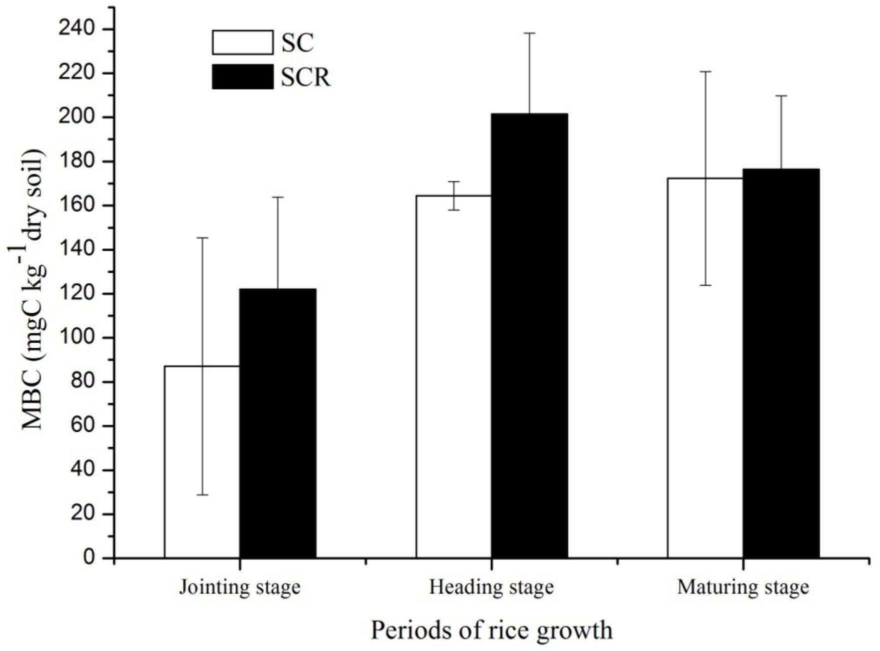

Fig. S2 Microbial biomass carbon (MBC) of soil during periods of rice growth

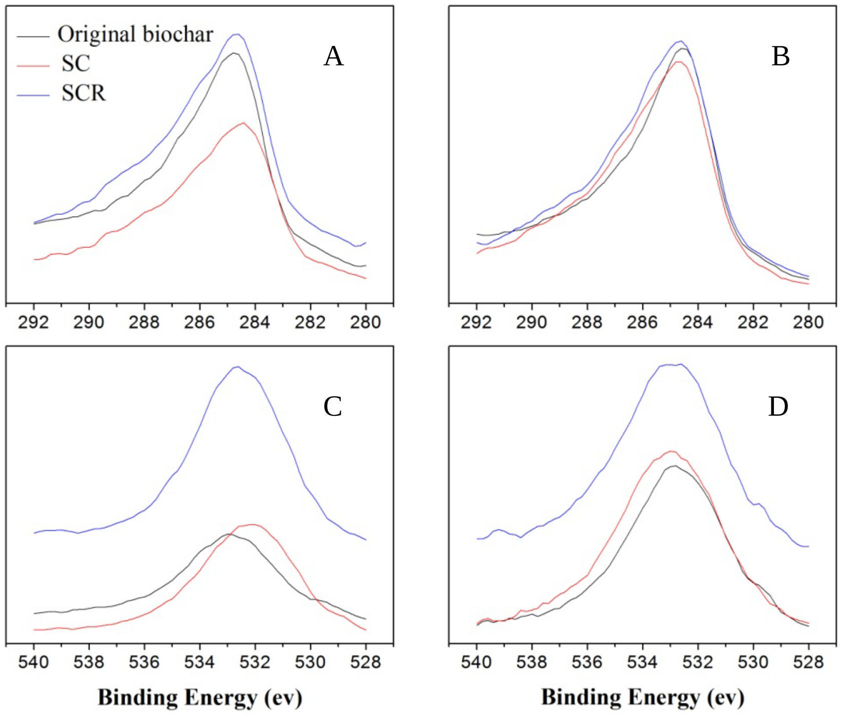

Fig. S3 X-ray Photoelectron Spectroscopy (XPS) spectra of the original biochar and biochar collected from SC and SCR treatments at the maturing stage of rice plant; C1s of biochar particles (A) and powder (B) as well as O1s of biochar particles (C) and powder (D)
